# Development and Characterization of Polylactic Acid (PLA)-Based Nanocomposites Used for Food Packaging

**DOI:** 10.3390/polym15132855

**Published:** 2023-06-28

**Authors:** Andrei Moldovan, Stanca Cuc, Doina Prodan, Mircea Rusu, Dorin Popa, Adrian Catalin Taut, Ioan Petean, Dorin Bomboş, Rami Doukeh, Ovidiu Nemes

**Affiliations:** 1 Department Environmental Engineering and Sustainable Development Entrepreneurship, Technical University of Cluj-Napoca, 400641 Cluj-Napoca, Romania; andrei_moldovan@mail.com; 2 “Raluca Ripan” Institute of Research in Chemistry, “Babes Bolyai” University, 400294 Cluj-Napoca, Romania; doina.prodan@ubbcluj.ro; 3 Lamar Auto Services S.R.L. Corpadea, 407038 Cluj-Napoca, Romania; mircea.a.rusu@yahoo.com; 4 Faculty of Exact Sciences and Engineering, “1 Decembrie 1918” University of Alba Iulia, 510009 Alba Iulia, Romania; dpopa@uab.ro; 5 Applied Electronics Department, Technical University of Cluj-Napoca, 400027 Cluj-Napoca, Romania; adrian.taut@ael.utcluj.ro; 6 Faculty of Chemistry and Chemical Engineering, “Babes-Bolyai” University, 11 Arany Janos Street, 400084 Cluj-Napoca, Romania; ioan.petean@ubbcluj.ro; 7 S.C. Medacril S.R.L., 8 Carpați Street, 551022 Mediaş, Romania; bombos.dorin@gmail.com; 8 Faculty of Petroleum Refining and Petrochemistry, Petroleum-Gas University of Ploiesti, 39 Bucharest Blvd., 100680 Ploiesti, Romania; rami.doukeh@yahoo.com

**Keywords:** polylactic acid, plasticizer, food packaging, physical-mechanical properties

## Abstract

The present study is focused on polylactic acid (PLA) blending with bio nanoadditives, such as Tonsil^®^ (clay) and Aerosil^®^, to obtain nanocomposites for a new generation of food packaging. The basic composition was enhanced using Sorbitan oleate (E494) and Proviplast as plasticizers, increasing the composite samples’ stability and their mechanical strength. Four mixtures were prepared: S1 with Tonsil^®^; S2 with Aerosil^®^; S3 with Aerosil^®^ + Proviplast; and S4 with Sabosorb. They were complexly characterized by FT-IR spectroscopy, differential scanning calorimetry, mechanical tests on different temperatures, and absorption of the saline solution. FTIR shows a proper embedding of the filler component into the polymer matrix and DSC presents a good stability at the living body temperature for all prepared samples. Micro and nanostructural aspects were evidenced by SEM and AFM microscopy, revealing that S3 has the most compact and uniform filler distribution and S4 has the most irregular one. Thus, S3 evidenced the best diametral tensile strength and S4 evidenced the weakest values. All samples present the best bending strength at 18 °C and fair values at 4 °C, with the best values being obtained for the S1 sample and the worst for S4. The lack of mechanical strength of the S4 sample is compensated by its best resistance at liquid penetration, while S1 is more affected by the liquid infiltrations. Finally, results show that PLA composites are suitable for biodegradable and disposable food packages, and the desired properties could be achieved by proper adjustment of the filler proportions.

## 1. Introduction

Food packaging material is one of the most widespread and important branches of the food industry because it assures the aliment’s integrity from the production line to the customer’s dishes; therefore, it has great importance in nutrition and human health. Food packaging provides a decisive condition in the final quality of the product, as it ensures protection against oxidation, biological, mechanical and/or physical modification, and contamination by external factors. There are a variety of packaging materials with different properties specific to each packaged product, including glass [1], transparent polyethylene terephthalate (PET) plastic [2,3], and paper [4], each with their respective advantages and disadvantages. Therefore, research in this field is very actual and has a lot of challenges to be explored.

A current requirement in this field is the use of renewable sources and raw materials that generate biodegradable packaging materials [5], assuring customers’ health protection, along with proper environmental behavior. One example is the use of polylactic acid as the main compound in recipes for packaging materials [3,6]. The advantages of this polymer are low production cost and non-toxicity compared to other raw materials used, and the major disadvantages are low barrier property and high fragility [7,8].

The properties of packaging materials, such as mechanical strength, permeability, and sealability, can influence their behavior regarding the purpose of food preservation and protection [9]. Testing of packaging materials generally involves the determination of physical and mechanical properties, assessing strength, stiffness, toughness, and impact properties, with a tendency for choosing accessible packaging materials [10]. Studying chemical stability is equally important to ensure product safety in the case of packaging materials for food-based applications [11].

The addition of nanoadditives to polymers in the composition of packaging mixtures improves their mechanical, optical, thermal, and barrier properties; subsequently, their use in the circular economy is more viable [12]. Moreover, the introduction of a quantity of 1–4% of fumed nanosilica led to a high thermal stability of the nanocomposites, presenting an improved processability [13]. In addition, silica nanoparticles (SiO_2_) can provide all these physico-mechanical properties [14], as well as antibacterial and antioxidant properties, to packaging materials [15,16].

Plasticizers in food packaging are additives used to increase the plasticity or decrease the viscosity of polymer blends, changing the physical properties of the packaging material, and increasing the flexibility of the packaging material by decreasing the attraction between polymer chains [17]. They have been shown to decrease the microhardness and Young’s modulus of polymer films by about 30%, yielding more flexible blends, with potential in the food packaging industry [18].

Several standards are developed for packaging materials testing, depending on each considered characteristic, such as, for example, ISO 178:2019-Plastics, referring to the determination of flexural properties. The European Union strategy for plastics in a circular economy was established in 2018 and provides specific guidelines regarding the physical, chemical, mechanical, and optical performance properties of the packaged product. Food packages also must consider the possibility of sudden temperature change to avoid fragilization caused by temperature drops, or local melting, in the case of high temperature exposure. Thus, the package braking, cracking, or melting is prevented and assures optimal protection of the contained aliments. Another demand of packaging is the protection of food from environmental conditions. So, the absorption of water or any other substance must be limited, providing the longest possible shelf life. On the other hand, water absorption can influence the degradation of the packaging material [19]. Literature data shows that absorption properties strongly influence the food package’s strength. Absorbed water can lead to degradation of the polymer structure, which implies package dimensional changes, with a significant weakening of their mechanical properties, and the extraction of water-soluble components from the exposed foods [20,21].

The objective of the work is the development and characterization of polylactic acid (PLA)-based nanocomposites to be used for food packaging. Current research is focused on the characterization of their physical and mechanical properties in certain conditions regarding safe food storage. The nanoadditives’ influence on the composite physical and mechanical properties follows. The main aim is to establish a classification of the possible application, regarding the obtained results at different temperatures, required by the food storage needs.

## 2. Materials and Methods

### 2.1. Materials

In this study, four mix recipes were obtained containing polylactic acid (by Nature Works LLC under the Ingeo^®^ brand, Tokyo, Japan), plasticizers, providing superior flexibility properties (Proviplast 2604, Proviplast 2624-by Proviron, Hangyhou, China), and fillers to improve the properties of the mixes (clay (Tonsil^®^–Sud-Chemia, Moosburg, Germany); Aerosil^®^-Degussa, Zurich, Germany; Sorbitan oleate (E494)-Ataman Kimya, Istanbul, Turkey) [22,23], (Table 1). Experimental PLA-based formulations were melt-processed on the Brabender Plastograph at a temperature of 180 °C, at 60 rpm, and with a mixing time of 15/30 min.

### 2.2. Samples Characterization

#### 2.2.1. Spectroscopic Characterization

The Fourier transform infrared (FT-IR) spectra of the obtained and investigated mixtures were recorded on the Jasco FTIR spectrometer (Jasco Europe srl, Cremella, Italy), equipped with a total attenuated reflectance (ATR) attachment with horizontal ZnSe crystal (Jasco PRO400S, Jasco Inc., Easton, PA, USA). The FTIR spectra were measured with a resolution of 4 cm^−1^, in the spectral range of 4000–500 cm^−1^, and the scans were repeated 100 times.

X-ray diffraction (XRD) was effectuated with an XRD-6000 Shimadzu Diffractometer produced by Shimadzu Company, Tokyo, Japan, using monochrome Cukα radiation with wavelength λ = 1.540560 Å. Aerosil^®^ powder was subjected to XRD investigation for proper evidence of its mineral characteristics. The pattern was registered from 2 to 90 degrees 2 theta, at a speed of 1 deg./min. It was analyzed with Match 3.0 software developed by Crystal Impact Company, Bonn, Germany.

#### 2.2.2. Evaluation of Thermal Characteristics by Differential Scanning Calorimetry (DSC)

Differential scanning calorimetry (DSC) was performed using the Mettler-Toledo 630e, 700 °C Calorimeter, Singapore. Measurement conditions for differential scanning calorimetry analysis were: aluminum crucible-40 μL; heating rate: 10 °C/min; temperature range 25–200 °C; end stage 0.5 min; atmosphere: nitrogen; flow rate: 80 mL/min.

#### 2.2.3. Mechanical Properties

The mechanical properties were studied using Lloyds Instron Universal Analyzer (Lloyd Instrumente, Ameteklns, West Sussex, England), equipped with a 5 kN cell. Ten specimens from each recipe were tested for each mechanical test.

##### Tensile Strength

The tensile tests were performed at room temperature using the dumbbell specimens (L × L × H = 40 × 4 × 3 mm^3^), according to the standard procedure UNE-EN ISO 527-3:2018 [24]. From the stress-strain curves, the elongation at fracture (E), tensile strength (TS), and Young’s modulus (E) of each mixture were calculated.

##### Flexural Strength

The flexural strength was measured using the three-point test, in accordance with ASTM D 790-03 [25]. Rectangular specimens (thickness = 4 mm, height = 4 mm, and length = 30 mm) were subjected to a three-point flexural force, with a distance of 20 mm between the two fixed points, and the mechanical test force being 0.5 N, at a speed of 0.5 mm/minute. In order to study the influence of transport and storage conditions on the packaging properties, specimens of each polymer blend were kept at two different temperatures for 24 h. After each exposure period, the test specimens were quickly transported in closed containers to the area for testing the flexural strength.

The tests were performed at:

✓ ambient temperature (24 °C), specific to the packaging used on the shelf;
✓ temperature of 4 °C (refrigerator temperature), for packages stored in refrigerated display cases; and
✓ temperature of −18 °C, as used in the refrigerated industry, for packaging frozen products.

#### 2.2.4. Absorption in Saline Solution

Liquid absorption is defined as the increase in weight of a sample, regarding its initial measured value, according to ASTM D570 [26]. Rectangular samples of 20 mm length, 10 mm width, and 3 mm thickness are placed in a desiccator at 23 °C until a constant mass value is obtained by weighing them with an analytical balance (Ohaus Explorer, Bucharest, Romania) with an accuracy of 0.001 g (initial M). The samples are further individually placed in solution with 15 mL of 10% saline solution at a constant temperature of 23 °C. At specific time periods (1, 4, 7, 13, 15, 18, 22 days), samples are removed from the immersion medium, carefully dried with a usual absorbent, and weighed (final M). The percentage of absorption is calculated with the formula:

(1) $$Ab=\frac{Mfinal-Minital}{Minitial}\times 100$$

Five specimens were prepared for each of the recipes investigated and were submitted until saturation of water absorption.

#### 2.2.5. Surface Analysis

##### Scanning Electron Microscope (SEM)

The surfaces of the samples were analyzed microscopically using the Inspect scanning electron microscope (FEI Company, Hillsboro, OR, USA) before and after the absorption determination, to determine surface changes following degradation in saline solution.

##### Atomic Force Microscopy (AFM)

The finest microstructural details and filler nanoparticles’ distribution within exposed composite samples were investigated by Atomic Force Microscopy (AFM). The investigation was carried out in Alternative Current (AC) mode on a Jeol JSPM 4210 Scanning Probe Microscope produced by Jeol Company, Tokio, Japan. Sample surface probing was effectuated using silicon nitride sharp sensors NSC 15 Hard types produced by MikroMasch Company, Sofia, Bulgaria. The sample surface was scanned at a square area with the side of 20 µm on at least five different macroscopic areas, chosen at random. The obtained topographic images were analyzed with Win SPM 2.0 Processing soft, Jeol Company, Tokio, Japan, displaying topographic images for morpho-dimensional observation and tridimensional profiles for roughness parameters measurements Ra and Rq.

*Ra* represents the arithmetic average of the profile height and is described by Equation (1) [27] and *Rq* represents the root mean square of the profile height and is described by Equation (2) [28]:

(2) $$R_{a}=\frac{1}{l_{r}}\int_{0}^{l_{r}}|z(x)|dx$$

(3) $$R_{q}=\sqrt{\frac{1}{l_{r}}\int_{0}^{l_{r}}|z{\left(x\right)}_{}^{2}|dx}$$

where: *l* is the profile length and *z* is the height at *x* point. Both *Ra* and *Rq* are important for various research applications.

#### 2.2.6. Statistical Analysis

Following the recording of the values obtained for each test, the differences within each group were subjected to statistical analysis using the Anova one-way test and the Tukey test (*p*-value below 0.05 being considered statistically significant) with the Origin2019b program. Mean and standard deviation were calculated for each group of results.

## 3. Results

### 3.1. Fourier Transform Infrared Spectroscopy (FTIR) Analysis

**Figure 1:**
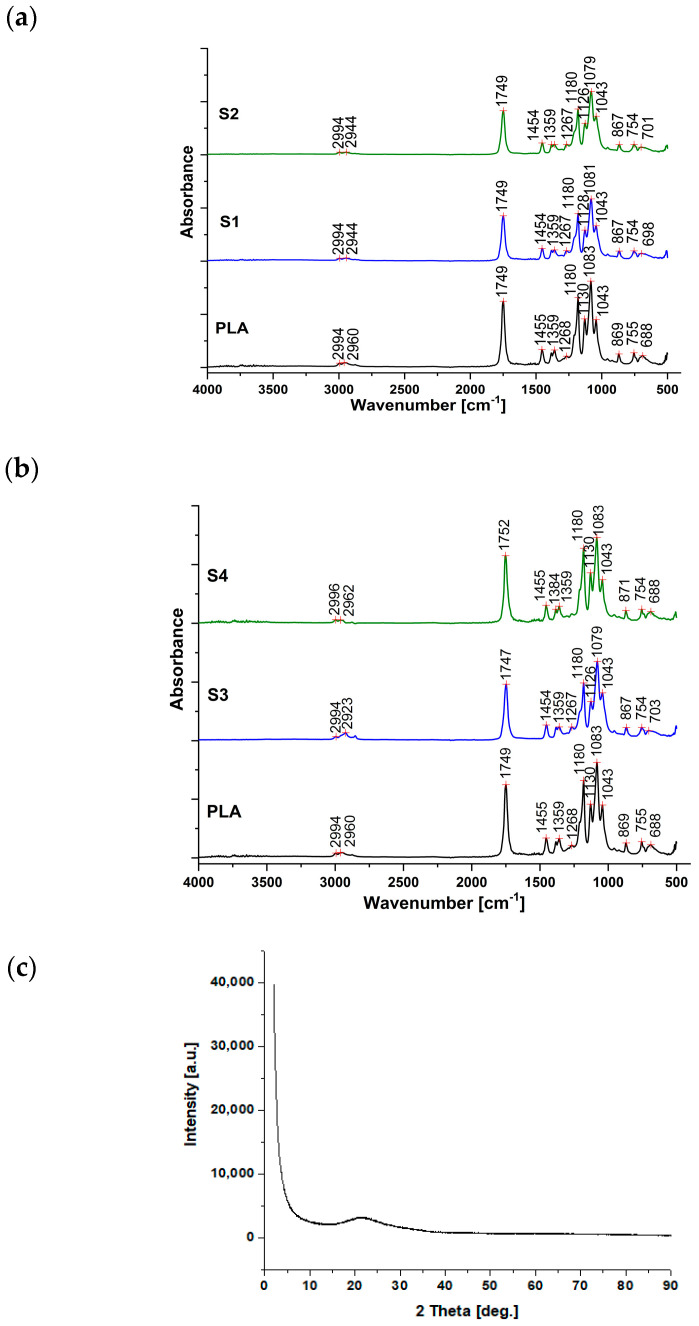
Spectroscopic investigation results: FTIR spectra for samples: (**a**) S1 and S2, (**b**) S3 and S4, and (**c**) XRD pattern result for Aerosil^®^ powder.

The absorption bands from 2994–2944 cm^−1^ correspond to the symmetric and asymmetric stretching of the CH_3_ group in all investigated samples, Figure 1a,b. The absorption band at 1749 (1752) cm^−1^ corresponds to the vibration of the C=O bond of PLA as a result of the presence of the ester [29]. This peak was intense due to the concentration of PLA and ester in the formulation.

The band at 1454–1455 cm^−1^ is attributed to the asymmetric CH_3_ bending vibration. The absorption band at 1361 (1359) cm^−1^ corresponds to the C-O-H group bending vibration. The band at 1267 (1268) cm^−1^ is associated with the C=O bending vibration, while the bands at 1180 and 1130 cm^−1^ correspond to the C-O-C stretching vibration of the ester groups [30]. Absorption bands at 1081 and 1043 cm^−1^, specific to the asymmetric stretching vibration of the Si-O-Si group in clay and Aerosil^®^, are observed. The spectra of 143 and 167 samples do not show a big difference among them, according to FTIR interpretation that is between the two types of Proviplast, 2604 and 2624.

Aerosil^®^ hydrophilic silica is a nanomaterial precursor of great applicative interest for polyester composites’ fabrication. A hydrophilic character and white color are the main advantages of Aerosil^®^ regarding polylactic acid-based food package manufacturing. Crystalline structures that might occur in such hydrophilic silica present a real danger of migration into the stored foods, which would result inconsequent contamination. Therefore, an amorphous mineral state of the hydrophilic silica is strongly required.

The XRD pattern for used Aerosil^®^ hydrophilic silica was registered in a wide-angle range for revealing all possible diffraction peaks, Figure 1c. The pattern allure shows a completely amorphous state of the Aerosil^®^ sample and no diffraction peaks were observed. The amorphous state of the sample was also proven by Match 3.0 analysis software. Thus, results show that the used hydrophilic silica is optimal for food packages.

A low density of Aerosil^®^ powder (≈2.2 g/cm^3^) diminishes sedimentation risk during polymeric charge melting. A high amount of amorphous silica results in a Lewis acidity of the processed composition; besides low water content, Brønsted acid favors super-acid formation at the polylactic acid charge melting temperature that might determine its reticulation and, consequently, improve its mechanical properties. A high porosity of Aerosil^®^ amorphous silica prevents volatile compound loss in additives used in food package manufacturing during the melting process.

### 3.2. Evaluation of Thermal Characteristics by Differential Scanning Calorimetry (DSC)

**Figure 2:**
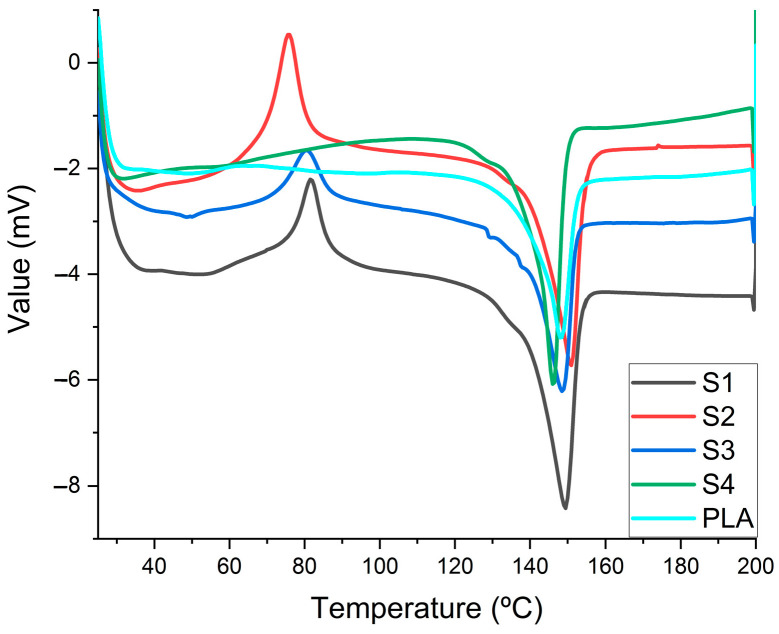
DSC curve of the samples investigated.

Differential scanning calorimetry (DSC) analysis is very effective on the thermal transition identification of the tested materials, which imply a rearrangement of the microstructural constituents as functions of the temperature variations [31]. Thus, Figure 2 evidences the thermal variation curves, featuring peaks that belong to the exothermic or endothermic reactions that occur in the tested samples.

The changes that take place in the pure PLA polymer, once the temperature increases, are two phase transitions: at 63.44 °C, where a maximum external crosslinking point is reached, and at 147.94 °C, with a maximum melting point.

DSC analysis of sample S1 revealed that plasticization in the presence of clay in polylactic acid with Proviplast 2604 favors the appearance of exothermic transformations at a temperature of 81.59 °C, and endothermic (probably softening) transformations at temperatures of 138.28 °C and 149.14 °C, respectively.

For sample S2, the DSC analysis revealed that the plasticization of polylactic acid with Proviplast 2604 in the presence of Aerosil^®^, at the same concentration as sample S1, favors the appearance of exothermic transformations at temperatures lower than 75.85 °C, and endothermic transformations (probably softening) at temperatures of 133.07 °C and 150.59 °C, respectively. For sample S3, with differences only of Proviplast 2624 from sample S1, DSC analysis detected an exothermic transformation at a temperature of 80.45 °C, and an endothermic transformation at 48.75 °C and 148.4 °C, respectively.

For sample S4, only endothermic transformation was favored at 128.99 °C and 145.91 °C, respectively, in the presence of E494. In this case, the exothermic transformation at 80–75 °C, present in the other three samples investigated, does not appear.

### 3.3. Mechanical Properties

#### 3.3.1. Tensile Strength

**Figure 3:**
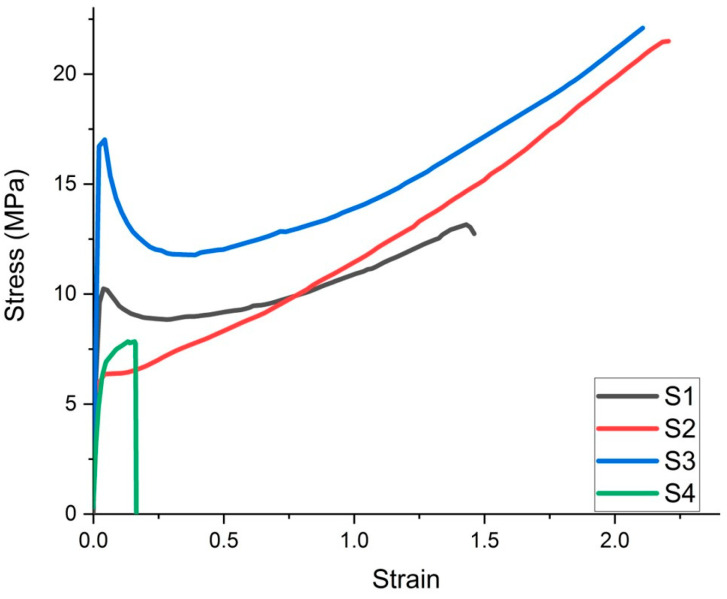
Deformation curve of investigated samples under tensile force.

Following the results obtained (Table 2), sample S3 showed the highest tensile strength, followed by S2. The lowest tensile strength values were recorded for sample S4. In terms of specimen elongation to failure, specimen S2 had a maximum elongation of 89 mm, followed by S3; the stiffest specimens with the lowest elongation were specimens S4, which also had the lowest stress at failure. It can be seen from Figure 3 that the mixtures with Aerosil^®^ give the highest strengths, followed by the specimens with clay in their composition (Figure 3).

The Anova test indicates a *p*-value ≤ 0.05; thus, it differs from the tested sample groups. Instead, the Tukey test gives statistical differences between all sample groups, in which each group is compared, in turn, with the other group; with the exception of the S2 and S3 groups, which are compared among themselves, for all tensile test determinations. The difference between the S2 and S3 samples is the different types of Proviplast (2604 and 2624), with the same ratio (15%), in the composition, but this is insignificant in terms of material characteristics under the influence of tensile forces.

#### 3.3.2. Flexural Strength

The flexural strength of the tested materials at room temperature varies from 20 MPa (S4) to 46 MPa (S1). The highest flexural strength is represented by the mixture of 3% clay and Proviplast 2604 (Table 3). Comparing the results of the room temperature flexural test between the four samples investigated, there are statistically significant differences between them (*p* ≤ 0.05) for all measured characteristics. The samples S1 and S2, which have no significant statistical differences, indicate that the flexural strength of the mixtures with clay is similar to those containing Aerosil^®^.

For the results of the flexural tests at refrigerator temperature, from 4 °C, both the strength and the Young’s modulus show semi-significant differences between the four samples investigated (Table 4). The Tukey test shows that, between samples S1 and S2, there are no differences for maximum flexural stress at maximum load and Young’s modulus. The decrease in temperature, for all the mixtures tested, caused an increase in both the elastic modulus and the maximum load supported, and a decrease in the elongation at break. These results are as expected, since lowering the temperature reduces the flexibility of the polymer chain and increases the stiffness of the material. However, the fluctuation of the differences in the bending strengths gave a different deformation curve, depending on the composition of the blends.

The statistical tests show that there are no significant differences between samples S2 and S3 in terms of strength and Young’s modulus of the samples analyzed, indicating that, at low temperatures, the different types of Proviplast (2604 and 2624) behave in the same way.

**Figure 4:**
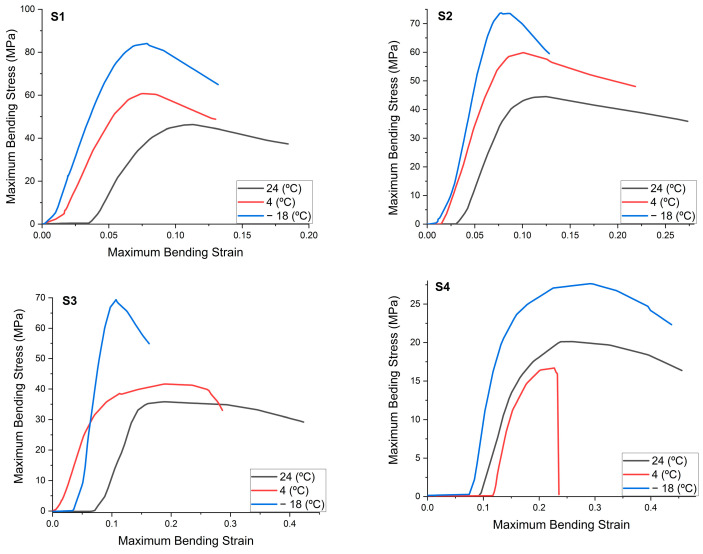
Deformation curve of samples as a function of storage temperature during the flexural test.

Evaluating the four samples during the flexural test according to the selected temperatures, we can say that sample S1 shows significant differences only in the group of samples analyzed at −18 °C, while, for the rest of the samples, there are differences between all of the three groups investigated at different temperature values (Table 5). It can also be seen from the deformation curves that samples S1 and S2 have a similar appearance: as the temperature decreases, the curve becomes sharper and narrower, a sign that the mixtures go from flexible and hard to rigid and brittle (Figure 4).

**Figure 5:**
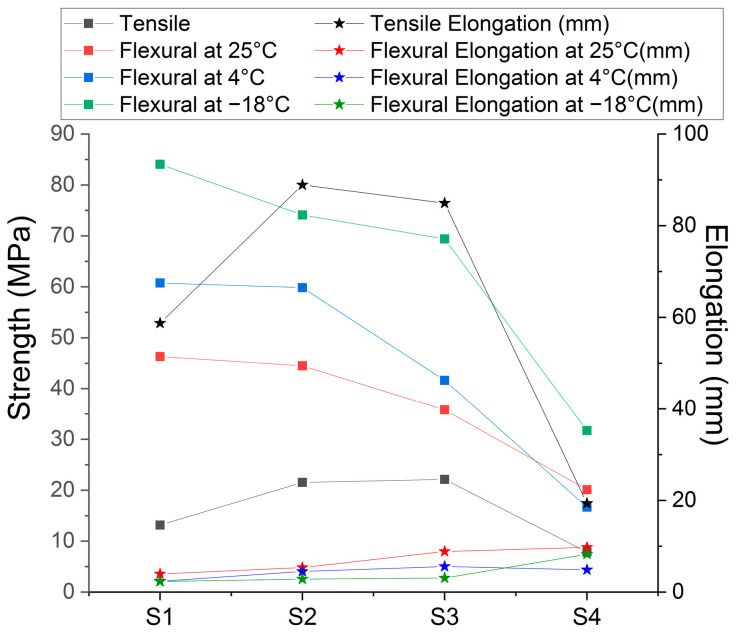
The behavior of the investigated samples under the action of traction or compression forces.

Figure 5 shows the results of the resistances obtained after the mechanical tests, depending on the composition of the investigated samples. All PLA nanocomposites with Proviplast showed a decrease in elongation under the action of temperature, due to the presence of rigid nanocrystals. The breaking strength reached a maximum for S2 and S3, when 3% Aerosil^®^ was added, resulting in the highest elongation of the samples.

### 3.4. Absorption of Saline Solution

The S4 sample does not present significant statistical differences between the absorption values in time, comparing the absorption values within each group according to the immersion time of the samples in water. This means that the immersion time does not influence the S4 sample’s reaction with water. The most stable sample in time, and compared to its initial stage, was S4.

**Figure 6:**
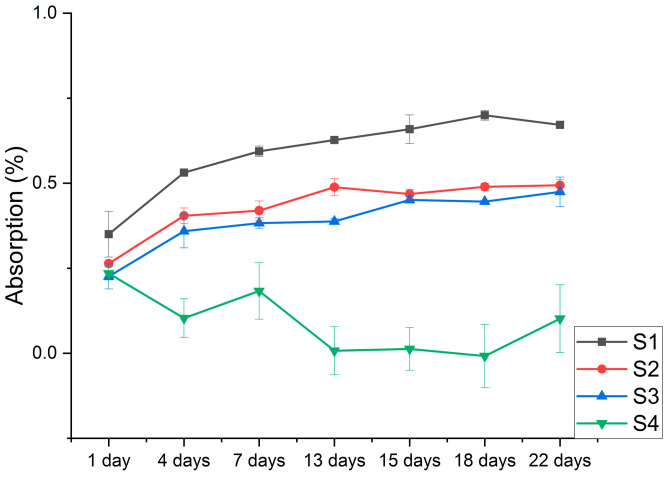
Absorption of the investigated mixtures as a function of immersion time.

Comparing the time evolution of water absorption for all investigated samples, there are significant differences between them (*p* = 8.82243 × 10^−5^). Following the Tukey test, statistical differences are seen between all the samples, in which each sample is compared in turn with the other samples in the group. As an exception, the S2 and S3 samples are compared among themselves, and the samples S3 and S4 are also compared among themselves (Figure 6).

Depending on the composition of the mixtures, between the S1 and S4 samples, where the difference is the addition of clay or E494, for both sample groups, the absorption values are higher on the first day of immersion and decrease over time to a constant value. The S2 and S3 samples, with different types of Proviplast, but with identical ratios of Proviplast in their composition, give the lowest absorption values.

### 3.5. Surface Analysis

**Figure 7:**
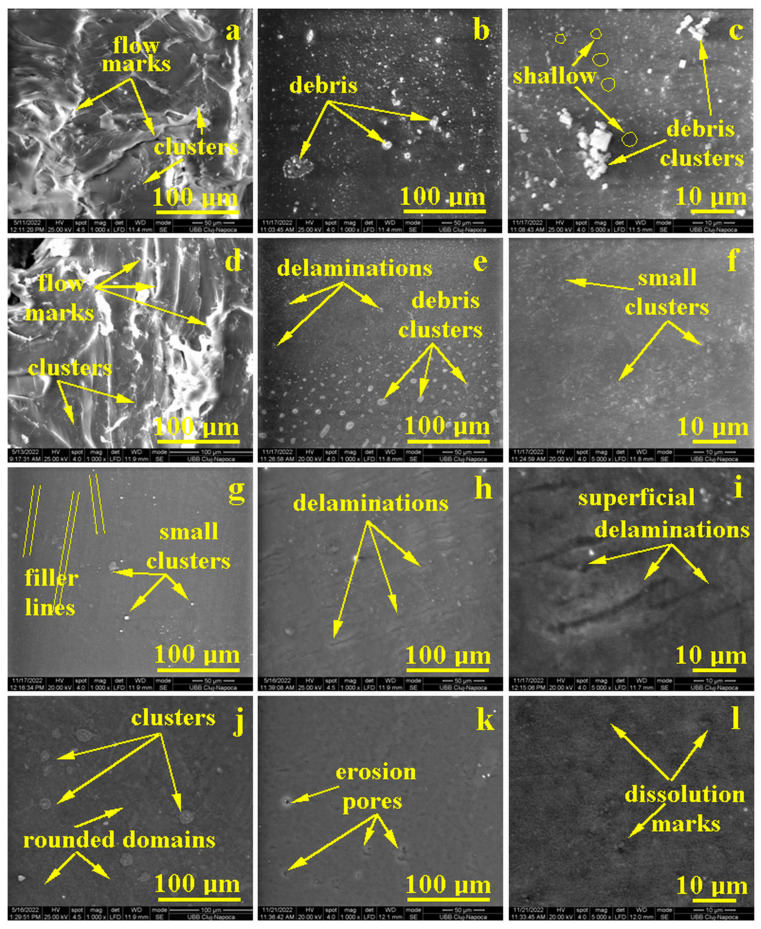
SEM images of the sample microstructure: (**a**) S1 initial, (**b**) S1 after exposure, (**c**) S1 after exposure–high magnification detail, (**d**) S2 initial, (**e**) S2 after exposure, (**f**) S2 after exposure–high magnification detail, (**g**) S3 initials, (**h**) S3 after exposure, (**i**) S3 after exposure–high magnification detail, (**j**) S4 initial, (**k**) S4 after exposure, and (**l**) S4 after exposure–high magnification detail.

The initial microstructure of sample S1, Figure 7a, reveals a complex mixture of PLA with Proviplast 2604 that acts as a plasticizer, which enhances the molding properties of the composite. This fact is observed by the polymer flowing marks having front lengths of about 150–200 µm and a width of 30 µm. The composite cohesion is assured, a fact confirmed by the pore’s absence, besides the apparently irregular surface. Clay filler is generally well-distributed into the composite bulk, but some fine microstructure clusters occur. These are local spots where clay particles are clogged together into rounded formations of about 2 to 5 µm, situated on the top of PLA-Proviplast mixture waves, Figure 7a.

The exposure to saline solution 10% initiates affects mainly the clay filler particles, due to the clay intense hygroscopic behavior factor, confirmed by the strong liquid absorption after just 1 day of immersion. The clusters situated in the outermost layer of the composite are subjected to the long-wet exposure during 22 days of storage, which contributes to their disaggregation, forming particulate debris, Figure 7b. The wavy aspect of the initial sample surface disappears due to the relative swelling of the nanofiller embedded into the polymer matrix, ensuring a compact microstructure. The microscopic detail in Figure 7c evidences the filler debris particles with boulder shapes, and sizes ranging from 2 to 5 µm. The polymer surface seems to be cohesive without local delaminations, with only some dark shallows with rounded shapes, which might be superficial pores formed by the bentonite clusters’ dissolution. This requires more enhanced microscopic investigation.

Sample S2 has a similar microstructure to the previous sample, due to the mixture of PLA and Proviplast 2604. It features the same polymer flowing waves, Figure 7d, with extended front lengths up to 250 µm, and a broader width of about 40–80 µm. The microstructural enhancement is caused by Aerosil^®^ filler nanoparticles, which are very well mixed onto the matrix. Only a few sporadic filler clusters are observed as small spots, ranging from 1–3 µm in size. This is explained by the high stability of the fumed silica particles, which are the base of Aerosil^®^ filler.

The polymer flowing waves within the S2 sample surface disappear after 22 days of saline solution exposure, due to the mild mineral loss of the exposed Aerosil^®^ nanoparticles, Figure 7e. Prolonged exposure to the liquid environment causes a local water infiltration between the exposed filler particles and the polymer matrix, a fact sustained by the slowly increasing water absorption observed in Figure 6. Hence, the water infiltration progresses, and some of the filler nanoparticles are completely delaminated from the matrix and form some clusters on the sample surface, Figure 7e, having rounded shapes, and sizes varying depending on their position in the image observation field.

On the top side, the clusters are very fine, and on the lower side of the images, their diameter is increased up to 3–5 µm. The area with small clusters was observed at higher magnification in Figure 7f. Some micron debris clusters are observed on the compact composite structure beneath them.

The S3 sample is plasticized with Proviplast 2624, which ensures a better homogenization and compactness of the microstructure, due to a better polymer flow during molding, Figure 7g. The more homogenous matrix embeds better the Aerosil^®^ nanoparticles, reducing considerably the number of agglomeration clusters (there are only few small spots below 3 µm in diameter). Thus, the mineral particles are better distributed onto the composite bulk, and the Aerosil^®^ particles are arranged in relative parallel lines due to the polymer flow during molding, Figure 7g, an aspect that should be revealed at a more enhanced microscopic observation.

The prolonged exposure (22 days) to the saline solution leads to delamination of exposed Aerosil^®^ nanoparticles from the outermost layer. Their dislocation from the composite surface generates small, elongated traces of about 30 µm lengths, oriented relatively parallel to one another. These are formed due to the progressive water infiltration, besides the exposed Aerosil^®^ nanoparticles and polymer matrix. Such behavior is in good agreement with the liquid absorption evolution observed in Figure 6. The microstructural detail in Figure 7i confirms the optimal distribution of Aerosil^®^ nanoparticles, ensuring a proper composite compactness that is unaffected by the liquid exposure to their deep layers.

The S4 sample contains Proviplast 2604, used as a plasticizer of the PLA base, and a small amount of 3% E494, which acts as an emulsifier. The emulsification of PLA/Proviplast polymer blend has beneficial effects on microstructure refinement. Figure 7j evidences a compact structure with rounded domains of about 1–3 μm, well-dispersed into the PLA base matrix, and several rounded clusters, ranging from 20–35 μm.

Long time exposure to saline solution (22 days) has a significant effect on the S4 sample surface, as observed in Figure 7k. The rounded clusters are significantly eroded, with small pores in their center. On the other hand, the fine, rounded domains uniformly dispersed into the PLA base present a good resistance to the liquid erosive effect. The microstructural detail in Figure 7l proves the good cohesion between the microstructural domains, ensuring an optimal compactness of the composite. This explains the liquid absorption behavior of this sample, which is almost constant, and the low value during the exposure time. The fine microstructure is slightly blurred due to the polymer dissolution attempt induced by the saline solution, but it properly resists deep penetration.

**Figure 8:**
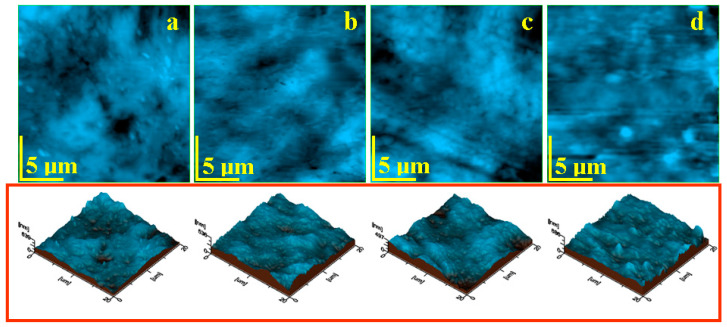
AFM topographic images of the samples after liquid exposure: (**a**) S1, (**b**) S2, (**c**) S3, and (**d**) S4. Tridimensional profiles are given below each topographic image.

The finest micro and nanostructural aspects regarding samples surfaces after 22 days of exposure to saline solution were revealed by atomic force microscopy images, Figure 8.

The topography of the S1 sample evidences the Tonsil^®^ nanoparticles well-embedded into the PLA/Proviplast polymer matrix, ensuring a proper cohesion of the material. Most of the bentonite particles within the clay filler are in the range of 60–80 nm, Figure 8a, but there are some bigger ones with slightly elongated shapes, due to their montmorillonite content (e.g., aluminum phyllosilicate mineral, which the base of bentonite consists of), with sizes of about 95–100 nm. Two rounded pores with irregular margins are observed: one is situated near the center of the image, and the other is in the upper right corner. They have rounded shapes with irregular margins and diameters between 2–5 μm, belonging to the former clusters observed in the SEM images. The liquid penetration of these clusters and local mineral loss causes the surface erosion. On the other hand, well-embedded bentonite nanoparticles swell at the liquid contact and increase the composite compactness, preventing further penetration of the liquid into the deeper layers. Therefore, AFM analysis indicates that Tonsil^®^ filler is effective against liquid penetration only if it is very well dispersed into the polymer matrix; otherwise, it is vulnerable, and might be easily destructured by the formation of large pores.

Aerosil^®^ filler is based on fumed silica nanoparticles, which are inert at contact with most of the erosive liquids, and 10% saline solution does not represent a concern regarding their integrity. Sample S2’s fine micro- and nanostructure, Figure 8b, reveals a very compact material, with Aerosil^®^ nanoparticles very well-embedded into the polymer matrix. It is relatively difficult to measure their exact sizes due to polymer embedding, but it is about 80 nm. The filler nanoparticle clusters are situated in the range of 1–3 μm, most of them being rounded, but elongated shapes might occur, especially due to the polymer flow during molding. The long-time exposure to saline solution (22 days) affects the sample surface by progressive liquid infiltration between Aerosil^®^ clusters and polymers, causing complete delamination. This fact is sustained by the pores observed in the surface, Figure 8b.

A better nanostructural embedding of Aerosil^®^ nanoparticles is observed for the S3 sample, Figure 8c. The Aerosil^®^ nanoparticles are very well-embedded into the polymer matrix in relatively parallel rows. This arrangement is caused by the composite flow during molding. The filler particle optimal embedding does not allow exact measuring of their diameter, but it looks about 80–90 nm. This sample has less cluster formation, but several Aerosil^®^ nanoparticles are situated on the outermost layer and are in direct contact with the saline solution. It progressively infiltrates at the silica/polymer interface, causing their loosening and, finally, their complete loss. This fact is evidenced by the demineralized areas situated on the left and right sides of the topographic image in Figure 8c. The nanostructure alteration observed by AFM is in good agreement with the SEM investigation.

Small micro-sized rounded domains within the S4 sample are better visualized in Figure 8d. Their size varies from about 800 nm to 3 μm. The topography evidenced a very good cohesion between polymer domains, ensuring a good uniformity of the composite bulk. However, the outermost layer is very blurred because of prolonged storage in the saline solution.

**Figure 9:**
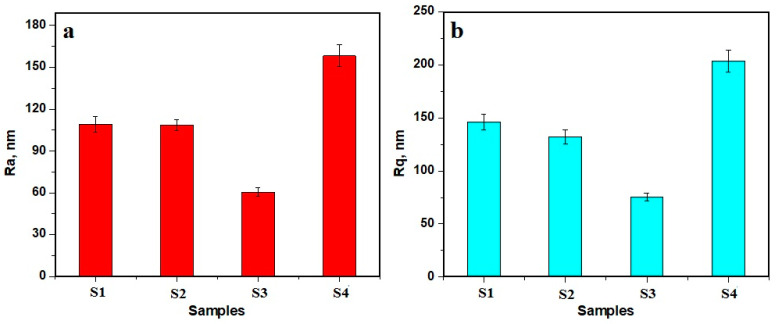
Mean surface roughness parameters measured with AFM: (**a**) Ra and (**b**) Rq.

Samples’ surface alterations, due to the exposure to the liquid, affect the roughness parameters. The average values of five determinations on the different macroscopic areas were determined to ensure a proper characterization, Figure 9.

The lowest average roughness was obtained for the S3 sample, due to the optimal dispersion of the Aerosil^®^ nanoparticles into the polymer matrix and its good resistance against in-depth penetration of the liquid, Figure 9. It is followed by the S2 and S1 samples, which had slightly increased values of roughness, due to the pore formations caused by the erosion of microstructural filler particle clusters. It is very interesting that the mean roughness of sample 201 is the greatest value obtained. This is a particular case that is not related to the mineral loss, but to the polymer surface corrugation, as a consequence of the long-term exposure to the saline solution.

## 4. Discussions

The plasticizers and additives investigated and added to PLA blends were used to improve the ductility, flexibility, and processability of PLA. There is a wide range of PLA blends of different types and production processes [32,33], which exhibit high mechanical strengths, but show brittleness and lower elongation at break (3%) [34,35]. These characteristics are compatible for packaging materials for products that need high protection, but, for films intended for food packaging, in addition to tensile strength, elongation must ensure resistance to pressures that occur during transport, application, and handling of packaged food [36,37].

Comparing the results of the blends obtained with pure PLA, the flexural (≈37 MPa) and tensile strengths (≈40 MPa) are lower than those investigated in the current study [38], but with a higher increase in elongation at break (by over 200%), which indicates that the material absorbs more energy before rupture occurs [39], a characteristic that also represents the flexibility of the material when stretched [40].

In 2009, Rhim [41] obtained PLA-based composite films with different types of nanoclays (5%), which managed to increase the elongation by up to 17%, but the tensile strength decreased to 40 MPa (20%) compared to pure PLA (50 MPa). Later, in 2013, Shirai et al. [42] obtained very high elongation values in the range of 72–148%, using different additions of adipate or citrate esters, but the tensile strength was below 1 MPa due to the incorporation of 70% starch. Since then, until today, a variety of blends have been analyzed and characterized to maintain the flexibility of the structure and increase the mechanical strength by adding additives and plasticizers, such as acetyl tri-n-butyl citrate (ATBC) [43], PLA with poly(hydroxybutyrate) (PHB) [44], cellulose [45], chitosan [46], lignan [47], poly(propylene carbonate) (PPC) [48], gelatin [49], and even essential oils [50].

The determination of the water absorption of the materials proposed for investigation in our study was carried out with the aim of understanding how the addition of some additives (plasticizers, clay, etc.) can influence the polymer matrix after their storage in a slightly saline solution for a set period. According to Table 1, all samples have the same percentage of PLA in their composition. Also, all investigated samples have the same percentage of Proviplast in their composition, with the mention that samples S1, S2, and S4 contain Proviplast 2604, and sample S2 contains Proviplast 2624. The samples also contain the same percentage of three more different ingredients, as the case may be (S1-clay, S2 and S3-Aerosil^®^, and S4-E494). After the first day of immersion in saline solution, the highest absorption value was recorded, in the case of sample S1 (1.75%), and the lowest value was recorded, in the case of sample S3 (0.22%). At the end of the investigation period, after 22 days, the highest absorption value was also recorded, in the case of sample S1 (0.67%), which was a much lower percentage than the one recorded after the first day of immersion. The lowest absorption value, at the end of the investigation period, was recorded in the case of sample S4 (0.10%).

Correlating all observed micro- and nanostructural aspects, it seems that the lack of mineral filler facilitates the resistance against liquid penetration. On the other hand, filler absence considerably reduces the mechanical properties of the samples. Therefore, a precise balance between composite constituents is required to gain the optimal mechanical properties, along with a good resistance to the liquid penetration.

## 5. Conclusions

The aim of this work was to modify the properties of PLA polymer blends by adding different additives (Tonsil^®^, Sabosorb, Aerosil^®^) as plasticizers (Proviplast) to obtain blends used in the food packaging industry.

DSC analyses revealed that both plasticizers and nanomaterials used in the preparation of polylactic acid receptacles influence the behavior, as well as the characteristics, of these receptacles. Also, the values of the operating parameters influence the transformation temperature and the type of processes (exothermic or endothermic) that take place during the transformation.

Tensile strength results indicated that the addition of 3% (by weight) Aerosil^®^ improves the deformability of PLA, increasing the strength up to two times more than Tonsil^®^ blends, and three times more than Sabosorb. In the case of the flexural test, subjected to the three-test temperatures, sample S1 proved to be the most thermally stable, and with the highest strengths. Moreover, the highest percentage of absorption in saline is also present in sample S1, due to the presence of Tonsil^®^, followed by the Aerosil^®^ and the Sabosorb sample (S4), which offers the smallest differences and percentages during immersion in saline solution, which indicates that the highest degree of degradation is offered by the S1 sample.

Therefore, changing the type of plasticizer (Proviplast 2604/2624) did not influence the properties of the investigated mixtures very much; on the other hand, the addition of nanoadditives, especially Tonsil^®^, presented a high flexibility and desirable mechanical and thermal resistance for their application in packaging manufacturing for single-use plastic products.

## Figures and Tables

**Table 1:** Composition of materials investigated.

wt %	Polylactic Acid	Proviplast 2604	Proviplast 2624	Tonsil^®^	Aerosil^®^	Sorbitan
Samples						
S1	82	15		3		
S2	82	15			3	
S3	82		15		3	
S4	82	15				3

**Table 2:** Results of the tensile strength test (Means ± Standard deviation).

Samples	Tensile Strength Test. (MPa)	Maximum Strength (N)	Breaking Strength (N)	Elongation at Fracture (mm)	Young’s Modulus (MPa)	Stress at Break (MPa)
S1	13.159 ± 2.742	128.43 ± 30.767	120.147 ± 27.51	58.75 ± 10.27	1030.65 ± 188.64	12.310 ± 7.084
S2	21.540 ± 7.411	220.52 ± 30.637	218.635 ± 29.97	88.90 ± 17.76	506.69 ± 125.24	21.356 ± 9.495
S3	22.148 ± 7.763	247.17 ± 27.228	246.091 ± 28.11	84.94 ± 12.14	1171.39 ± 97.78	22.051 ± 3.285
S4	7.864 ± 2.144	77.86 ± 16.542	13.991 ± 15.84	19.35 ± 6.37	189.22 ± 20.54	3.309 ± 1.663

**Table 3:** Results of the flexural test at 24 °C (Means ± Standard deviation).

Samples	Maximum Load (N)	Young’s Modulus at Flexural (MPa)	Flexural Stiffness (Nm^2^)	Maximum Flexural Stress at Maximum Load (MPa)	Maximum Bending Stress at Maximum Load	Elongation (mm)
S1	68.377 ± 12.35	1201.78 ± 212.23	0.0138 ± 0.009	46.32 ± 2.135	0.113 ± 0.09	3.972 ± 1.21
S2	79.360 ± 19.95	956.13 ± 198.22	0.0145 ± 0.008	44.51 ± 5.01	0.125 ± 0.08	5.359 ± 2.01
S3	58.197 ± 13.51	634.75 ± 380.14	0.0083 ± 0.001	35.82 ± 2.34	0.187 ± 0.08	8.871 ± 1.95
S4	30.362 ± 6.28	346.83 ± 168.28	0.0040 ± 0.001	20.12 ± 0.10	0.259 ± 0.09	9.781 ± 2.13

**Table 4:** Results of the flexural test at 4 °C (Means ± Standard deviation).

Samples	Maximum Load (N)	Young’s Modulus at Flexural (MPa)	Flexural Stiffness (Nm^2^)	Maximum Flexural Stress at Maximum Load (MPa)	Maximum Bending Stress at Maximum Load	Elongation (mm)
S1	127.714 ± 13.61	1421.56 ± 161	0.0274 ± 0.009	60.78 ± 9.06	0.074 ± 0.031	2.389 ± 0.58
S2	98.644 ± 21.62	1211.23 ± 190	0.0162 ± 0.002	59.86 ± 1.17	0.100 ± 0.041	4.516 ± 1.67
S3	73.122 ± 11.90	628.93 ± 179	0.0094 ± 0.001	41.64 ± 3.02	0.188 ± 0.026	5.583 ± 1.74
S4	26.265 ± 9.06	491.77 ± 98	0.0061 ± 0.001	16.69 ± 1.03	0.227 ± 0.059	4.875 ± 0.59

**Table 5:** Results of the flexural test at −18 °C (Means ± Standard deviation).

Samples	Maximum Load (N)	Young’s Modulus at Flexural (MPa)	Flexural Stiffness (Nm^2^)	Maximum Flexural Stress at Maximum Load (MPa)	Maximum Bending Stress at Maximum Load	Elongation (mm)
S1	169.990 ± 31.73	1879.84 ± 201.55	0.0342 ± 0.010	84.08 ± 5.49	0.078 ± 0.019	2.332 ± 0.98
S2	101.182 ± 11.13	1711.81 ± 150.31	0.0175 ± 0.008	74.12 ± 1.92	0.077 ± 0.025	2.854 ± 0.75
S3	126.465 ± 23.69	1976.76 ± 210.86	0.0312 ± 0.010	69.40 ± 0.41	0.107 ± 0.033	3.080 ± 0.99
S4	59.581 ± 18.68	609.43 ± 192.90	0.0102 ± 0.007	31.73 ± 1.50	0.274 ± 0.028	8.268 ± 1.25

## Data Availability

Not applicable.
